# Idiopathic Nephrotic Syndrome in Children in Chad: Epidemiology and Clinical Outcomes

**DOI:** 10.3390/jcm12247626

**Published:** 2023-12-11

**Authors:** Guillaume Mahamat Abderraman, Youssouf Djidita Hagré, Hissein Ali Mahamat, Senoussi Charfadine, Ali Sakine Amne, Adoum Attimer Khadidja, Lionel Rostaing

**Affiliations:** 1 Nephrology-Dialysis Department, CHU la Renaissance, N’Djamena, Chad; zalba2001@yahoo.fr (G.M.A.); nephrochad@gmail.com (H.A.M.); scharfadine@gmail.com (S.C.); 2 Paediatric Department, CHU Mère et Enfant, N’Djamena 2029, Chad; djiditi20@yahoo.fr (Y.D.H.); amnealisakine@gmail.com (A.S.A.); dockady@yahoo.fr (A.A.K.); 3 Nephrology, Hemodialysis, Apheresis and Transplantation Department, CHU Grenoble-Alpes, 38700 La Tronche, France

**Keywords:** nephrotic syndrome, pediatrics, nephrology, steroids, FSGS, Chad, sub-Saharan countries

## Abstract

Introduction: Nephrotic syndrome (NS) remains the most common presentation of glomerular diseases in children. Moreover, NS is primarily idiopathic, accounting for 90% of cases, with an average onset age between 2 and 10 years. The objective of our study was to describe the characteristics and outcomes of NS in children from three major hospitals in one of the world’s poorest countries, Chad. Patients and Methods: This observational, cross-sectional, descriptive, and multicenter study took place over a period of 36 months (1 January 2019–31 December 2021) and was carried out in three hospitals in N’Djamena, Chad. Children aged 1–15 years presenting with NS were included in the study. Results: Out of 16,776 children hospitalized or followed up with in outpatient clinics, 24 cases of NS were identified, yielding a prevalence of 0.14%. The median age at presentation was 6.16 years (1–10). Nineteen children were male (sex ratio 3.8). Eight cases were classified as impure NS (33.3%). Edema was present in all patients, while oliguria was present in 29.16% (*n* = 7), and arterial hypertension was present in 20.83% (*n* = 5) of cases. Mean proteinuria, albuminemia, and total proteins were 2.86g/L, 19.13g/L and 30.41g/L, respectively. The median serum creatinine was 87.3 µmol/L (75–1375 µmol/L). Three patients experienced acute renal failure upon admission. Four patients had secondary NS. All idiopathic NS patients (*n* = 20) who had received corticosteroid therapy had a 90% response rate to steroids. Non-responsive or relapsed patients underwent kidney biopsy (*n* = 7), revealing focal segmental glomerulosclerosis (FSGS; *n* = 4) as the most common histological lesion, followed by minimal change disease (*n* = 2) and membranoproliferative glomerulonephritis (*n* = 1). The median length of hospitalization stay was 10.67 (5–27) days. None of the patients with idiopathic NS died. At the last follow-up, sixteen patients (80%) achieved long-term complete remission with normal renal function; however, four of those had subsequent relapses. One patient with secondary NS died. Conclusion: In Chad, childhood idiopathic nephrotic syndrome predominantly affects young males; steroid sensitivity is as high as 95%, and in the long-term, 80% of patients achieve remission with normal renal function.

## 1. Introduction

Postinfectious glomerulonephritis, Henoch–Schönlein purpura nephritis, and minimal change disease (MCD) remain the most common causes of glomerular disease in children and can be diagnosed clinically without the need for a biopsy [1]. IgA nephropathy is the most common pediatric glomerular disease diagnosed by kidney biopsy and is considered the most common chronic glomerulopathy worldwide [1]. Childhood nephrotic syndromes result mostly from two idiopathic diseases: minimal-change nephrotic syndrome and focal segmental glomerulosclerosis (FSGS) [2]. Conversely, membranous nephropathy is rare in children, and other causes of isolated nephrotic syndrome are either rare genetic disorders or secondary diseases associated with drugs, infections, or neoplasia. Nephrotic syndrome (NS) is defined by nephrotic-range proteinuria (≥40 mg/m^2^/h, urine protein/creatinine ratio ≥200 mg/mL, or 3+ protein on urine dipstick), hypoalbuminemia (<25 g/L), and edema. The incidence of idiopathic nephrotic syndrome is 1.15–16.9 per 100,000 children, varying by ethnicity and region. Even though the cause of idiopathic NS remains unknown, its pathogenesis is thought to involve systemic circulating factors, inherited podocyte structural abnormalities, or immune dysregulation. Genetic risk is mostly described among children with steroid-resistant disease [3]. The cornerstone of therapy is prednisone for most patients who are steroid-sensitive (i.e., steroid-responsive) [4,5]; however, the disease can run a frequently relapsing course, necessitating alternative immunosuppressive agents such as cyclosporine, tacrolimus, cyclophosphamide, and rituximab [6,7]. The main complications of NS are infections and venous thromboembolisms, as well as a risk of acute kidney injury. The prognosis in terms of long-term kidney outcome is excellent for steroid-responsive disease, and steroid resistance is an important determinant of the future risk of chronic kidney disease (CKD) or end-stage kidney disease (ESKD).

A comprehensive retrospective study conducted at a large single center assessed the outcomes of 372 children diagnosed with idiopathic NS, comprising focal segmental glomerulosclerosis (FSGS: 57%), minimal change disease (MCD: 20.6%), and diffuse mesangial proliferation (21.9%). The study followed these patients for a minimum of 5 years after diagnosis [8]. Of the cohort, two hundred and ninety-nine of the patients (80.4%) exhibited a positive response to steroids, while seventy-three (19.6%) did not. Steroid sensitivity was notably higher in patients with MCD and in those under the age of five years. Conversely, resistance to steroids was most prevalent in children with FSGS. Complete remission was achieved by 96% of steroid-sensitive patients and 46.6% of those resistant to steroids. Fifteen percent of steroid-resistant patients developed chronic kidney disease. Drawing from the PodoNet Registry, which tracked 1354 children with steroid-resistant NS, 612 of whom had documented responsiveness to intensified immunosuppression (IIS), the study identified that initial IIS responsiveness and the identification of a hereditary podocytopathy were indicative of favorable and poor long-term outcomes, respectively [9].

Nowadays, in tropical Africa, children with NS survive more often than before. This is probably related to improved access to healthcare, as well as a shift in clinical patterns favoring idiopathic NS and increased sensitivity to corticosteroids [10]. Nephrotic syndrome is currently the leading cause of chronic kidney disease and end-stage kidney disease in children from sub-Saharan Africa. Apart from limited reports from Nigeria and Sudan, the overall incidence of NS in Africa is unknown [11]. In Chad, a study conducted in 2012 on nephrotic syndrome in children in the pediatric department of the Mother and Child Hospital of N’Djamena reported a prevalence of 1.36% [12]. Ten years after that study, we decided to conduct a multicenter study across the three main hospitals in N’Djamena (Chad) with the objective of describing the characteristics and outcomes of pediatric idiopathic nephrotic syndrome in one of the poorest countries in the world (https://hdr.undp.org/data-center/human-development-index#/indicies/HDI, accessed on 1 September 2023). The goal of the study was to investigate how idiopathic nephrotic syndrome in children was managed in one of the poorest countries in the world.

## 2. Patients and Methods

Settings of the study:

The study was conducted in three hospitals in N’Djamena, Chad, namely the pediatric department of the “Mother and Child” Hospital and University Center, the “Chad-China Friendship” Hospital, and the Nephrology department of the “Renaissance” Hospital and University Center. This was an observational, cross-sectional, descriptive, and multicenter study conducted over a 36-month period from 1 January 2019 to 31 December 2021.

Population: 

From the medical records of 16,776 children who were either hospitalized or followed up in outpatient clinics, we identified 24 children with NS aged between 1 and 10 years. NS was defined by proteinuria greater than 50 mg/kg/d, hypoalbuminemia less than 30 g/L, and hypoproteinemia less than 60 g/L [13]. NS is considered impure when associated with at least one of the following elements: hematuria, arterial hypertension, or non-selective proteinuria (albuminuria < 85%) with or without (acute) renal failure. Those with idiopathic NS (*n* = 20) were placed on steroid therapy (prednisone 2 mg/kg/d for 4 weeks, then a taper of 2 mg every 2 days for 2 months until 60 mg/d, then 45 mg/d for 2 weeks, then 30 mg/d for 2 weeks, and finally 15 mg/d for the last 2 weeks).

Definition of corticosensitivity: 

Corticosensitivity is defined by complete remission within 4 weeks of the administration of prednisone/prednisolone at the standard dose (60 mg/m^2^/day or 2 mg/kg/day, maximum 60 mg/day [14]). Children who do not show complete remission of proteinuria following 4–8 weeks of treatment with corticosteroids are considered to have steroid-resistant nephrotic syndrome. Steroid dependence is defined by a relapse during the decrease in steroid therapy or within less than three months after stopping it. 

As the children were managed according to international guidelines, ethical committee approval was not necessary. However, for every child, the parents had to give written informed consent to use their children’s data.

Data were gathered and collected from patient records using a pre-established questionnaire. The information was entered into Excel 2016 software and analyzed using Sphinx V5 software. The results were presented in terms of frequency and percentage for qualitative variables and means with their standard deviation for quantitative variables. In cases where appropriate, the medians (ranges) were calculated. The analytical study utilized cross tables. A comparison of means with their standard deviation and percentages was conducted using Student’s test and a Chi-squared test, based on their respective conditions of applicability. The significance threshold was set at “*p*” < 0.05.

## 3. Results

Among a total of 16,776 children hospitalized in pediatrics or followed up in the outpatient clinic during the study period, 24 cases of nephrotic syndrome were recorded, representing a prevalence of 0.14%. The median age at diagnosis was 6.16 (1–10) years. Nineteen children (79%) were male; i.e., there was a sex ratio of 3.8. Sickle cell disease background (heterozygosity) was present in 12.52% (*n* = 3) of cases. The average patient weight was 22.6 kg (11–42), and the average height was 113.21 cm (131–145). Arterial hypertension was observed in 20.9% (*n* = 5). Furthermore, 91.7% of patients exhibited an alteration in general status. The average diuresis was 1983.3 mL/24 h (range: 2001–3500), with oliguria being found in 33.3% (*n* = 7) of cases. All patients presented with lower limb edema and proteinuria on the urine dipstick exceeding 2++ (Table 1). Edemas were associated with pericarditis in 54.1% of cases and with anasarca in 28.1% of cases. Concerning biological parameters, the median serum creatinine was 87.3 µmol/L (75–1375.1 µmol/L), with abnormalities being noted in three children. The mean proteinuria was 2.86 g/d (range: 2.17–3.90). Protidemia was less than 40 g/L in 20 patients (83.3%). 

**Figure 1:**
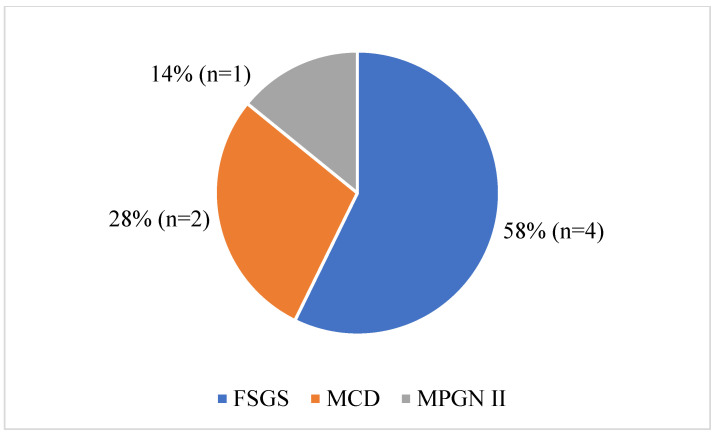
Renal pathology findings in seven patients who had a kidney biopsy. Abbreviations: FSGS, focal segmental glomerulosclerosis; MCD, minimal change disease; MPGN, membranoproliferative glomerulonephritis.

Fifty percent of the patients exhibited albuminemia levels between 10 and 20 g/L; in one patient, serum albumin was <10 g/L, while in eleven patients (55%), it ranged between 20 and 30 g/L. Hypocalcemia, defined as corrected calcemia < 88 mg/L, was observed in seven patients (29.1%). Prerenal acute renal failure was noted in two patients (8.3%). Regarding urine analysis, 12.5% of patients (*n* = 3) had leukocyturia, and 4.1% (*n* = 1) had hematuria in the Addis count. Upon admission, four children (16.6%) were diagnosed with Escherichia coli urinary tract infection (identified through a cytobacteriological examination of urine). Serum complement testing was conducted in only one patient, a 7-year-old girl presenting with NS, hematuria, and hypertension. Low levels of C4 and CH50 fractions were found, along with the presence of anti-factor H and factor I autoantibodies. Renal ultrasounds revealed enlarged kidneys in eight children (33.3%). NS was impure in six patients (25%). A kidney biopsy was performed in seven patients (29.1%), prompted by the persistence of impure nephrotic syndrome (hematuria and hypertension) in the setting of steroid dependency in two patients (8.3%), steroid resistance in two children (8.3%), and frequent relapses in three cases (12.5%). Our histological analysis (Figure 1) indicated that focal segmental glomerulosclerosis (FSGS) was the most common lesion, as it was observed in four children (16.6%), followed by two cases (8.2%) of minimal change disease (MCD), and one case (4.1%) of membranoproliferative glomerulonephritis (MPGN). In four children (16.6%), nephrotic syndrome was not idiopathic, including two cases of sickle cell nephropathy, one case of atypical hemolytic uremic syndrome, and one case of Burkitt’s lymphoma. Regarding therapy, the 20 patients with idiopathic NS received corticosteroid therapy based on prednisone (as mentioned above). The average length of hospitalization was 10.67 days, with extremes ranging from 5 to 27 days. The median duration of steroid therapy was 114 days (range: 62–155). 

**Figure 2:**
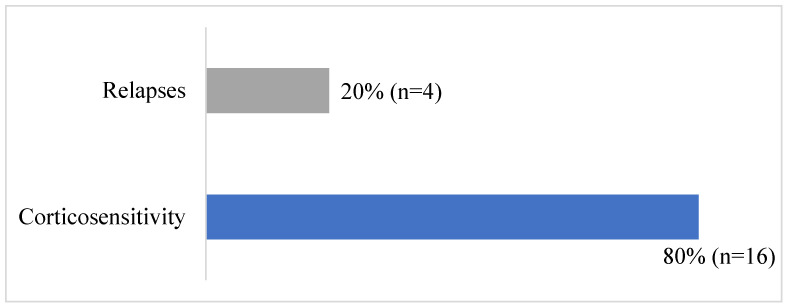
Patients’ outcomes following treatment for idiopathic NS.

Table 2 and Figure 2 summarize patients’ outcomes. Eighteen patients achieved complete remission (90%). However, two patients had one to two relapses per year with normal renal function at last follow-up, and two patients had more than three relapses per year, progressing to CKD stage 3a. In the end, long-term remission with normal renal function was achieved in sixteen patients (80% of the cohort). Unfortunately, one patient died, specifically the one with secondary NS due to atypical hemolytic and uremic syndrome. Their death occurred amidst rapidly progressive acute renal failure and sepsis. 

## 4. Discussion

There are very few studies addressing idiopathic nephrotic syndrome in children in sub-Saharan countries. Our study is one of the largest, and it demonstrates steroid sensitivity in idiopathic NS in 90% of cases, with long-term remission in 80% of them.

In 2014, a study reviewed the documents of all children presenting with renal diseases between January 2010 and December 2012 in two major pediatric hospitals in the Niger Delta region of Nigeria, namely the university hospital in Oghara and the GN Children’s Clinic in Warri. They found that renal diseases (*n* = 110) accounted for 1.6% of all admissions during the same period. Out of 110 patients, there were 73 males (66.3%), and about half of the patients were aged 5 to 10 years old. The most common presentations were nephrotic syndrome (30%), acute glomerulonephritis (18.2%), urinary tract infection (16.3%), acute kidney injury (10.9%), chronic kidney diseases (7.3%), and obstructive uropathy (7.3%). Regarding the renal disease outcomes, 80% of the patients achieved full recovery, and unfortunately, 14.6% died [15]. Conversely, in the same country, Lapedo et al. reported that kidney diseases accounted for 8.9% of pediatric admissions, with a prevalence of 22.3 admissions per 1000 child admissions per year. Nephrotic syndrome, acute kidney injury, and nephroblastoma accounted for almost 70% of admissions. The overall mortality rate was 14.4%, with acute kidney injury accounting for 36% of deaths [16].

In 2016, Muoneke et al. reported on a single-center study from Nigeria. Over a 3-year period, 1780 children were admitted, of which only 4.4% (79/1780) had renal disorders. They found that nephrotic syndrome was the most common cause of these disorders, being present in 32.9% of cases (26/79). The association between treatment mode and the outcome of the treatment was statistically significant (*p* = 0.03) [17].

In 2016, Kebede Mola et al. reported on a single-center study from Ethiopia. Out of 14,521 pediatric ward admissions during the study period, kidney diseases accounted for 473 admissions in 381 children, constituting 3.3% of all admissions. The three most common renal diseases observed were congenital anomalies of the kidney and urinary tract (CAKUT)—seen in 127 children (26.8%)—followed by nephrotic syndrome in 80 children (16.9%) and acute glomerulonephritis in 58 children (12.2%). Out of 381 children, 207 (54.3%) recovered normal renal function, 20 (5.2%) still had proteinuria, 13 (3.4%) progressed to chronic kidney disease, and 11 (2.9%) died. Additionally, 61 nephrotic children (76.3%) achieved remission, but 17 children (21.3%) still had proteinuria, and one steroid-resistant NS child died of end stage renal disease [18].

From these studies, it can be concluded that in sub-Saharan countries, the prevalence of renal disorders in children admitted in pediatric departments ranges from 1.6 to 8.9%. However, we do not yet have any data regarding the prevalence/incidence of idiopathic nephrotic syndrome in sub-Saharan countries. It has been shown that the epidemiology and treatment of childhood nephrotic syndrome in most of North Africa, as well as among White and Indian populations in South Africa, closely resembles that of European and North American populations. However, in the past, secondary causes of nephrotic syndrome (e.g., hepatitis B-associated nephropathy and quartan malaria nephropathy) were predominant among black people in Africa. Over time, the proportion of secondary cases has decreased, along with the rates of steroid resistance. However, among patients with steroid resistance, focal segmental glomerulosclerosis has been increasingly reported [19]. Recently, among 209 black children from Nigeria, it was found that the proportion with steroid-sensitive NS was comparable to proportions described in children of Asian and European descent. In addition, from that cohort, it was observed that children with steroid-sensitive NS (85.9% of the study cohort) had lower serum creatinine and higher glomerular filtration rates than those with steroid-resistant NS [20]. Moreover, among those aged 0–5 years, 92.6% had steroid-sensitive NS compared with 69.2% in those aged 11–17 years at the time of diagnosis. Finally, it was observed that the proportion of children with steroid-sensitive NS increased from 73.8% between the years 2010 and 2012 to 88.4% afterwards [20]. The same group reported on 108 children (median age: 5.9 years, peak: 1–2 years) with NS (from January 2008 to April 2013), 90.2% of whom had idiopathic nephrotic syndrome [20]. Nephrotic syndrome was diagnosed based on the following: 24 h urine protein > 40 mg/m^2^/h or spot urine protein, creatinine ratio > 200 mg/mmol, hypoalbuminemia (serum albumin < 25 g/L), generalized oedema, and hypercholesterolemia (serum cholesterol > 5.2 mmol/L). They found that the median time to remission was 7 days, and that steroid sensitivity was 82.8% among children with idiopathic nephrotic syndrome but 75.9% overall. As compared with steroid-resistant patients, those who were steroid-sensitive had a median age that was significantly lower. Finally, the predominant histologic finding in resistant cases was focal segmental glomerulosclerosis (53.3%). No cases of hepatitis B virus nephropathy or quartan malaria nephropathy were diagnosed. Overall mortality was 6.5% [21].

In our study, we observed a male prevalence (79% of the cohort, sex ratio 3.8). Similar male prevalence has been reported in other studies, such as a male–female ratio of 1.4 in Nigeria [20], Ivory Coast [22], and Algeria [23].

Renal-type edema was present in all our patients (100%). Consistent findings were reported by Ndongo et al. [24] and Mabiala-Babela et al. [25] in their respective studies. The presence of edema represents a prominent and nearly ubiquitous clinical manifestation of childhood nephrotic syndrome, constituting the primary reason for medical consultation.

Regarding idiopathic NS, we observed that nearly all patients (90%) exhibited steroid sensitivity. This percentage surpasses rates reported in other regions, such as Sudan (66%) [26], Ethiopia (73.3%) [18], Senegal (77%) [14], or Nigeria, where rates ranged from 82.8 to 85.9% [20,21]. However, at the last follow-up, we found that only 80% of the patients achieved remission with normal renal function. A study from Ethiopia reported a more prolonged persistence of a high rate of proteinuria [18] in a significant proportion of patients (21.3%) in the long term.

At the last follow-up, we observed that 80% of patients with idiopathic NS maintained remission. Conversely, the remainder exhibited either steroid dependency or resistance. This prompted kidney biopsies for 7 out of our 20 patients. Among them, six cases revealed histological lesions, with two cases presenting FSGS lesions (*n* = 4), and two cases showing MCD lesions (*n* = 2). Correspondingly, Ladapo et al. found FSGS to be the predominant histological lesion in Nigerian children with steroid-resistant NS [21]. Studies from Algeria and Sudan have reported FSGS lesions in 57.4% [27] and 44% [26] of cases, respectively. It is acknowledged that individuals with FSGS lesions are at risk of evolving to CKD; for instance, children failing to achieve remission within 5 years face a 50% risk of advancing to end-stage renal disease, whereas those who attain complete remission exhibit a 5-year kidney survival rate of 90% [28].

The main strength of our study lies in its comprehensive coverage of three principal hospitals in Chad, capturing all cases of idiopathic NS over a recent 3-year period. Uniform management protocols were applied across all cases. Furthermore, kidney tests were consistently performed for those exhibiting steroid dependency or resistance. However, financial constraints limited our capacity to conduct an extensive array of laboratory tests, constituting a noteworthy limitation of this study.

## 5. Conclusions

The prevalence of childhood idiopathic nephrotic syndrome remains inadequately explored in sub-Saharan countries. Within this cohort of 24 children diagnosed with nephrotic syndrome in Chad, 20 cases were identified as being idiopathic. Notably, steroid sensitivity reached 90% among those with idiopathic NS, and over the long term, 80% of the patients achieved remission with normal renal function.

## Figures and Tables

**Table 1:** Socio-demographic and clinical characteristics at diagnosis.

Variable	*n* = 24 Patients	Frequency (%)
**Gender**		
Female	5	21
Male	19	79
**Background**		
Sickle cell disease	3	12.5
**Clinic**		
Proteinuria	24	100
Hematuria	6	25.0
Leukocyturia	3	12.5
Lower limb edema	24	100
Pericardial effusion	13	54.17
Anasarca	7	29.17
Ascitis	9	37.5
Pleural effusion	3	12.5
Infiltration of the external genitals	4	16.7
Oliguria	8	33.3
Hypertension	5	20.8

**Table 2:** Patients’ outcomes following treatment for idiopathic NS (20 patients).

	Number of Patients (*n*)	Frequency (%)
Long-term remission with normal kidney function	16	80
1–2 relapses per year with regular monitoring in nephrology and normal renal function	2	10
>3 relapses per year with evolution towards chronicity (eGFR < 60 mL/min)	2	10
Total	20	100

## Data Availability

Data are available upon reasonable request.

## Abbreviations

NS, nephrotic syndrome; FSGS, focal segmental glomerulosclerosis; MCD, minimal change disease; CKD, chronic kidney disease; ESKD, end-stage kidney disease.
